# Achieving the Millennium Development Goal for Under-five Mortality in Bangladesh: Current Status and Lessons for Issues and Challenges for Further Improvements

**DOI:** 10.3329/jhpn.v29i2.7813

**Published:** 2011-04

**Authors:** Amir Mohammad Sayem, Abu Taher Md. Sanaullah Nury, Md. Delwar Hossain

**Affiliations:** ^1^Bangladesh Institute of Social Research, Zigatola, Dhanmondi, Dhaka 1209, Bangladesh; ^2^Upazila Family Planning Office, Narayanganj Sadar, Bangladesh; ^3^Foreign Exchange Department, Bangladesh Bank, Dhaka 1000, Bangladesh

**Keywords:** Achievements, Child mortality, Infant mortality, Millennium Development Goals, Review literature, Bangladesh

## Abstract

The study assessed the achievements in, critically reviewed the relevant issues of, and put forward recommendations for achieving the target of the Millennium Development Goal relating to mortality of children aged less than five years (under-five mortality) in Bangladesh within 2015. To materialize the study objectives, a thorough literature review was done. Mortality of under-five children and infants decreased respectively to 65 from 151 and to 52 from 94 per 1,000 livebirths during 1990-2006. The immunization coverage increased from 54% to 81.9% during the same period. The projection shows that Bangladesh will achieve targeted reduction in under-five mortality and infant mortality within the time limit, except immunization coverage. Neonatal mortality contributed to the majority of childhood deaths. Contribution of neonatal mortality to child mortality was the highest. There were remarkable differences in child mortality by sex, division, and residence. To progress further for achieving the target of MDG relating to child mortality, some issues, such as lower use of maternal healthcare services, hazardous environmental effects on childhood illness, high malnutrition among children, shorter duration of exclusive breastfeeding practices, various child injuries leading to death, low healthcare-use of children, probable future threat of financial shortage, and strategies lacking area-wise focus on child mortality, need to be considered. Without these, the achievement of MDG relating to child mortality may not be possible within 2015.

## INTRODUCTION

One hundred eighty-nine countries attending the United Nations Millennium Summit signed the United Nations Millennium Declaration in 2000 to eradicate extreme poverty, hunger, and diseases among one billion people in the world, who subsist barely on anything (1). This project set a deadline of 2015 to achieve eight goals, known as Millennium Development Goals (MDGs). One of the goals was to reduce child mortality by two-thirds by 2015 from 1990. Evidence suggests that, region-wise, East Asia, Southeast Asia, Latin America/Caribbean countries, and Europe are on track to achieve the MDG 4 but South Asia was described as still having high mortality, and the target of MDG 4 may not be met by 2015 (2). The Government of Bangladesh and the United Nations Country Team in Bangladesh expressed concerns at the slowing of the pace in the decline of mortality of children aged less than five years (under-five mortality) (3). The most recent nationally-representative data in Bangladesh indicate that Bangladesh has made significant improvements in reducing the child mortality rate, although it is far below the MDG target. Child mortality per 1,000 livebirths came down from 94 in 1990 to 52 in 2007 (4); however, the decline has been faster in urban areas than in rural areas (5).

A very successful family-planning programme and the remarkable progress in expanding child-immunization coverage are mainly responsible for the decline in child mortality in Bangladesh. Different NGOs and private clinic/hospitals, especially in urban areas, played a vital role for the higher decline in urban areas than in rural areas. However, challenges still remain as there are higher neonatal mortality rates, higher child malnutrition, and differences regarding mortality by sex, division, and residence in Bangladesh (4). With the right types of interventions, the child mortality-related MDG may not be very difficult to achieve.

This paper focused on the available evidence of current health status of children, causes and proximate determinants of child morality, health-related programmes, and financial challenges to achieve the MDGs relating to under-five mortality. The objectives of the study were to present the achievement status, provide a critical review of relevant issues, and put forward recommendations for achieving the target of MDG relating to under-five mortality within 2015.

## CHILD MORTALITY IN BANGLADESH

### Levels and trends

The decline of under-five mortality in some developing countries, including Bangladesh, was striking in recent years (6). Based on the indicators set for the attainment of MDG 4 (Table 1), Bangladesh is moving slowly towards the targets set in relation to the reduction of child mortality. Data showed that, in all three indicators, Bangladesh, to a greater extent, is on the track in achieving the MDG relating to child mortality (Table 1). For example, under-five mortality decreased from 151 in 1990 to 65 per 1,000 livebirths in 2006, with a rate of 5.38 per year against the required annual rate of 2.14 per year. The projection shows that, if the present rate sustains, it would have been achieved the target at the end of 2009. Almost a similar decrease was found in infant mortality which seems to achieve the target at the end of 2015. However, the immunization coverage is still lagging behind, to some extent, from the annual required rate of achievement. It increased from 54% in 1990 to 81.9% in 2006, with the yearly achieved rate of 1.74% against the yearly required rate of 1.84%. The projection suggests that Bangladesh may achieve the target of immunization coverage in 2017, if the current rate of increase can be sustained in the coming years. However, to achieve the MDG relating to under-five mortality within the time limit, consideration should be given on causes of child mortality and its proximate determinants. Although, statistically, Bangladesh is going to achieve its target for child health within the time limit, except immunization, further achievement depends on many challenging factors, including sociocultural, programmatic and financial barriers.

**Table 1.** Targets of MDG, achievements, required achievements per year, and projected year of achievement on reduction of child mortality in Bangladesh

Global targets	Bangladesh targets	Indicator	Base year (1990)	Current status (2006)	Target in 2015	Achieved per year*	Required annual achievement since 1990†	Projected year of achievement‡
Reduce under-five mortality rate by two-thirds between 1990 and 2015	Reduce under-five mortality rate from 151 deaths per 1,000 livebirths in 1990 to 50 by 2015	Under-five mortality rate	151	65	50	Decreased 5.38	Decrease 2.14	2009
		Infant mortality rate	94	52	32	Decreased 2.63	Decrease 2.50	2015
		Proportion of 1-year old children immunized against measles	54%	81.9%	100%	1.74% increased	1.84% increased	2017

Source of base-year data: Millennium Development Goals: Bangladesh progress report, 2005 (3)

Source of current status data: Bangladesh Demographic and Health Survey, 2007 (4)

*Calculated by subtracting the current from the base status, and the result is divided by 16 (1990-2006);

†Calculated by subtracting target from the base status and dividing the result by 25 (1990-2015);

‡Calculated by subtracting the current and target status, dividing by achieved rate per year and adding results to 2006; MDG=Millennium Development Goal

Data of the latest Bangladesh Demographic and Health Survey showed that infant, child and under-five mortality are respectively 52, 14, and 65 per 1,000 livebirths (Table 2) (5). Data further showed that, in 2007, under-five mortality in developing, least-developed, South African and South Asian countries were respectively 73, 131, 134, and 78 per 1,000 livebirths. It indicates that under-five mortality in Bangladesh is relatively lower. Neonatal mortality largely contributed to most under-five mortality (37 of 52) in this country. So, reduction of under-five mortality largely depends on reduction of neonatal mortality in Bangladesh. It necessitates the importance of focusing on neonatal health more than other child mortality indicators. Data also showed that there are variations in child mortality, by sex, division, and residence. For example, male children experienced higher rates of mortality in all aspects of child mortality (e.g. neonatal mortality, postneonatal mortality, infant mortality, and under-five mortality), except child mortality. Sylhet division has the highest mortality rates for all-mortality indicators, except child mortality. Infant mortality ranges from 50 deaths per 1,000 livebirths in Barisal to 84 per 1,000 livebirths in Sylhet. Child mortality is the highest in Chittagong. Under-five mortality is the lowest in Khulna (58 per 1,000 livebirths) while it is the highest in Sylhet (107 per 1,000 livebirths). Khulna has the lowest rates of infant, child and under-five mortality while Barisal has the lowest rates of neonatal mortality. Postneonatal mortality is the lowest in Rajshahi division. The sharp difference was also found in child mortality, by residence, except postneonatal mortality which was 17 for urban and 18 for rural areas. Data showed that 81.9% of children received immunizations in 2006. Although the sex difference was negligible, the differences by division and residence are clear (Table 2).

**Table 2.** Child mortality and immunization by sex, division, and residence in Bangladesh

Characteristics	Neonatal mortality*	Postneonatal mortality†	Infant mortality‡	Child mortality¶	Under-five mortality§	Immunized**
Sex						
Male	42	19	61	16	76	81.2
Female	36	17	54	20	72	82.5
Division						
Barisal	31	19	50	23	71	90.2
Chittagong	34	20	54	27	79	77.2
Dhaka	38	18	55	14	69	82.4
Khulna	32	17	49	10	58	88.9
Rajshahi	46	12	58	14	71	85.6
Sylhet	53	31	84	25	107	70.8
Residence						
Urban	33	17	50	13	63	86.3
Rural	41	18	59	19	77	80.5
Total	37	15	52	14	65	81.9

Source of data: Bangladesh Demographic and Health Survey 2007 (4)

*The probability of dying within the first month of life per 1,000 livebirths;

†The difference between infant and neonatal mortality per 1,000 livebirths;

‡The probability of dying before the first birthday per 1,000 livebirths;

¶The probability of dying of a child between the first and the fifth birthday per 1,000 children surviving to their first birthday;

§The probability of dying between birth and the fifth birthday per 1,000 livebirths;

**Children who took the required number of vaccines, such as BCG, DPT, polio, hepatitis, and measles

### Causes of child mortality

The figure shows that acute respiratory infection (ARI) is the leading cause of under-five mortality in Bangladesh and is responsible for 21.1% of deaths. Birth asphyxia, premature birth, or low birthweight (LBW), and diarrhoea are also, respectively, the important causes of 11.7%, 6.5%, and 5.1% of under-five mortality in Bangladesh. Congenital abnormality, neonatal tetanus, and malnutrition are, to some extent, responsible for child morality in the country. Data from the latest BDHS also showed the higher prevalence of ARI (28.1%) and diarrhoea (9.8%) in Bangladesh. The prevalence of diarrhoea is slightly higher among boys, among children living in Chittagong, Sylhet, and Dhaka divisions, and in urban areas (4). Rural children are more likely to suffer from ARI than urban children. Higher proportions of children living in Sylhet, Rajshahi, and Chittagong divisions have symptoms of ARI than those in other divisions (4).

### Proximate determinants of child mortality

Various medical and non-medical causes of child mortality are influenced by many socioeconomic, cultural, political and environmental factors. Although the factors are diverse, the well-known analytical framework by Mosley and Chen (1984) offers a scheme that treats socioeconomic factors, such as individual productivity of fathers and mothers, income/wealth, ecological setting, political economy, and the health system, as the independent variables that must act through five proximate determinants (8). The proximate determinants are: (a) maternal factors in the reproduction process, (b) environmental contaminations, (c) nutritional deficiency, (d) injuries to the child, and (e) practices in healthcare of the child. A critical review of the situations against the proximate determinants of child mortality and some relevant issues in Bangladesh is presented below.

Various maternal factors are responsible for deaths of children. Babies born to young mothers are more likely to be premature, have LBW, and have a higher risk of child mortality (9, 10). If all births occurring within less than two years of each other could be more widely spaced, one in four infant deaths in developing countries might be prevented (11). Data showed that antenatal service uptake is low in Bangladesh. Antenatal care (ANC) is essential for both maternal and child health. The risk of maternal mortality and morbidity and neonatal deaths can be reduced substantially through regular and proper ANC check-ups and deliveries under safe and hygienic conditions (12, 13). Data further showed that a birth interval of less than two years was found among 15.1% of women in Bangladesh (4). The median age of women at first birth in Bangladesh is only 19 years that put women and children at risk of death. Medical attention at the time of delivery, ANC, and place of delivery (14) are important determinants of child survival in Bangladesh. However, 60% of women received ANC at least once from any care provider while around 52% received care from a medically-trained care provider (Table 3). Nationally, although over 90% of women are protected against tetanus, 9.8% are still not covered while, division-wise, the differences are considerable (highest 93% in Rajshahi and lowest 82.4% in Sylhet). Only 15% of births in Bangladesh take place at a health facility with significant urban-rural and divisional differences. The scenario is the worst in Sylhet and Barisal where only 8.2% and 9.5% of deliveries respectively take place at health facilities. Less than one-fifth (18%) of deliveries are assisted by a medically-trained care provider (MTP). The lower maternal service uptake in Sylhet may be due to several factors, including religious influence, superstitions, and lower awareness while, in contrast, such factors are less influential in Dhaka.

**Table 3.** Use of maternal healthcare service in Bangladesh by division and residence

Characteristics	Received ANC	ANC by MTP	TT injection	Delivery at health facility	Delivery by MTP	Postnatal care (women)
Division						
Barisal	52.3	43.0	89.4	9.5	13.4	17.6
Chittagong	60.3	52.0	86.9	13.6	18.5	23.5
Dhaka	56.8	47.8	91.6	16.9	19.8	22.4
Khulna	60.9	51.0	92.8	22.4	26.5	28.4
Rajshahi	64.6	54.5	93.0	13.2	15.4	17.3
Sylhet	54.4	45.7	82.4	8.2	10.9	16.2
Residence						
Urban	75.7	71.1	92.3	30.6	36.7	39.0
Rural	56.4	45.8	89.6	10.5	13.2	16.5
Total	60.3	51.2	90.2	14.6	18.0	21.3

Data source: Bangladesh Demographic and Health Survey 2007 (4).

ANC=Antenatal care;

MTP=Medically-trained care provider;

TT=Tetanus toxoid

**Fig.**
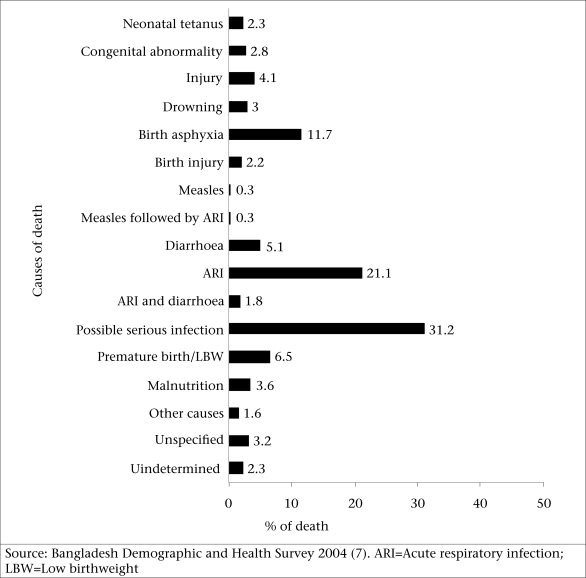
Causes of death of under-five children in Bangladesh

Data showed that access to an improved source of drinking-water is universal (97%) in Bangladesh (4). Tubewells are the most common source of drinking-water in both urban (69%) and rural areas (96%) but 93% of households do not treat drinking-water with significant differences by residence. Households without proper sanitation facilities have a greater risk of diseases, such as diarrhoea, dysentery, and typhoid, than households with improved sanitation facilities. Overall, one in four households has an improved toilet facility (flush-toilet or pit-latrine with slab). This problem is more common in rural areas where 10% of households have no toilet facilities compared to 2% in urban areas. About nine in 10 households use solid fuels in Bangladesh, and this proportion has declined little (from 93% to 91%) since 2004. Virtually, as cost is involved, the most socially-disadvantaged groups may not have much access to the protective effect of hygienic or sanitary latrines.

In Bangladesh, 43.2%, 17.4%, and 41% of under-five children are, respectively, considered to be stunted, wasted, and underweight (Table 4). There is a little difference in stunting between male and female children. Rural children are more stunted than urban children (45% vs 36%). The rate of stunted children is lower in Khulna (35%) and higher in Barisal (47%). Dhaka has a lower proportion of wasted children than all other administrative divisions. Other background differentials for wasting are similar to the differentials in stunting but smaller. The percentage of underweight children was higher in rural areas compared to urban areas. The lowest (34.1%) acute underweight was recorded in Khulna division while the highest (45.6%) was recording in Barishal division. Technical intervention should focus on malnutrition, infectious and parasitic diseases, and immunizations delivered through a strengthened basic healthcare system. Malnutrition contributes to over half of child deaths in Bangladesh, and the low birth rate is estimated to affect 30-50% of infants.

**Table 4.** Nutritional and breastfeeding status of children by sex, division, and residence in Bangladesh

	Nutritional status			Breastfeeding status			
Characteristics	Stunted (acute)	Wasted (acute)	Underweight (acute)	Ever breastfed	Colostrum received	Median duration of any breastfeeding	Median duration of exclusive breastfeeding
Sex							
Male	43.7	18.4	39.9	97.6	92.1	32.0	1.5
Female	42.7	16.5	42.1	97.9	92.3	33.3	2.2
Division							
Barisal	46.9	18.0	45.6	96.9	90.9	31.0	0.7
Chittagong	45.5	17.6	41.6	97.1	88.6	25.6	2.9
Dhaka	44.0	15.4	39.9	98.1	94.1	35.8	1.0
Khulna	54.6	18.8	34.1	98.3	95.9	36.3	2.0
Rajshahi	41.8	19.1	43.3	98.2	94.4	37.0	1.2
Sylhet	44.7	18.3	42.1	97.1	83.8	28.0	0.7
Residence							
Urban	36.4	8.5	33.4	98.1	94.0	29.5	1.6
Rural	45.0	12.7	43.0	97.7	91.7	34.5	1.8
Total	43.2	17.4	41.0	97.8	92.2	32.8	1.8

Source of data: Bangladesh Demographic Health Survey 2007 (4)

The median duration of breastfeeding among Bangladeshi children is 32.8 months whereas the median duration of exclusive breastfeeding is only 1.8 months (Table 4). The median duration of any and exclusive breastfeeding varies little across the background characteristics. However, any breastfeeding is the longest among children living in Rajshahi and Khulna divisions (37 months and 36 months respectively) and the shortest in Sylhet and Chittagong divisions (28 months and 26 months respectively). The differentials in the median duration of exclusive breastfeeding are small, except that Chittagong division stands out as having the highest median duration of exclusive breastfeeding (2.9 months). Rural and female children were more likely to be breastfed by any and exclusive breastfeeding compared to urban and male children of Bangladesh.

Injuries among young children are recognized as a major public-health problem in early childhood (15, 16). In Bangladesh, injury is one of the leading causes of death of children aged 1-4 years. It was estimated that nearly half a million deaths occurred due to drowning in 1998 globally, 57% of which occurred among children (17). A study found that maternal characteristics were strongly and independently associated with an increased risk of mortality from injuries during early childhood: low education, young age, and increased number of children. The association was similar for both infants and children aged 1-4 years (18). Childhood injuries take different forms in Bangladesh, such as drowning, burning, falling down from bed or higher places, etc. There are few studies on injury-related mortality in Bangladesh. A study in Matlab on drowning found that drowning-associated deaths were significantly higher among children aged 1-4 years (19). The same study also reported that around 60% of deaths due to drowning occurred in ponds and ditches, which are situated around the victims’ households. Drowning remains an important and preventable cause of child mortality, and adequate preventive strategies are absent.

Proper healthcare reduces child mortality and morbidity but the use of heathcare by children is poor in Bangladesh. Postnatal check-up detects health problems and provides healthcare for early childhood diseases. Data showed that only 21% of children received it with significant urban-rural and divisional differences (Table 5). Vitamin A, an essential micronutrient for the immune system, reduces blindness, along with the severity of infections, such as measles and diarrhoeal diseases in children. Supplementation of vitamin A covers 88.3% but it is not equally distributed by sex, division, and residence. Although diarrhoea is one of the leading causes of child mortality in Bangladesh, one in five children with diarrhoea was taken to a medically-trained healthcare provider for advice or treatment. Female children, urban children, and children living in Khulna were more likely than other children to be taken to a health professional or a health facility for the treatment of diarrhoea. Over 80% of children use oral rehydration solution (ORS) or home-made solution (HMS) in response to diarrhoea. This is a major achievement in Bangladesh compared to other least-developed countries. However, variations in using ORS or HMS have been observed in divisions where Rajshahi had the highest and Sylhet had the lowest use-rate. Zinc for the treatment of diarrhoea has been shown to decrease subsequent morbidity due to diarrhoea and acute lower respiratory infection in older children but not among infants aged less than six months (20, 21). However, only 2.5% of children with diarrhoea are treated with it. Thirty-seven percent of children with symptoms of ARI were taken to a health facility or to a medically-trained care provider for treatment. Boys are more likely than girls to be taken to a health facility or MTP when ill with ARI. Significant urban-rural and divisional differences were also observed from the BDHS data. Over 12% of children do not receive any kind of treatment for ARI.

**Table 5.** Healthcare of under-five children by sex, division, and residence in Bangladesh

Characteristics	Post natal check-ups	% given vitamin A supplements in past 6 months	Treatment for diarrhoea			Treatment for ARI	
			Health facility	ORS or homemade solution^*^	Zinc syrup/tablets	Health facility	No treatment
Sex							
Male	NA	88.5	16.5	85.2	1.8	40.0	11.6
Female	NA	88.1	24.1	85.2	3.3	32.0	13.9
Division							
Barisal	20.0	84.9	23.6	87.2	2.5	45.1	26.8
Chittagong	24.4	86.0	18.9	77.9	5.2	34.4	13.5
Dhaka	22.5	89.7	17.3	90.0	1.9	37.1	8.9
Khulna	30.7	90.7	30.4	87.9	0.0	-	-
Rajshahi	17.2	88.8	17.3	90.8	1.9	35.9	11.1
Sylhet	16.4	87.5	23.8	73.2	0.8	33.0	17.0
Residence							
Urban	40.0	90.3	27.4	85.5	1.7	56.6	14.1
Rural	17.0	87.8	17.7	85.1	2.7	33.3	12.4
Total	21.9	88.3	19.8	81.2	2.5	37.1	12.7

Source of data: Bangladesh Demographic Health Survey-2007 (4)

ORS=Oral rehydration solution

NA=Not available

## LESSONS FOR IMPROVEMENTS

The main focus should be given on neonatal mortality. It should be noted that most births and neonatal deaths in developing countries occur in the home, without the assistance of healthcare professionals (22). Despite the importance of the pattern of practices in newborn care, little is known about routine care in the home and the impact on neonatal outcomes. It particularly occurs in cultural contexts such as in Bangladesh, where mothers and infants are secluded from others for a certain time after birth. Previous studies on home-care practices in Bangladesh have focused principally on delivery issues (23), largely overlooking many routine practices that may significantly impact the health and survival of newborns. Outreach care, health education to improve home-care practices, recognition of danger-signs, generation of demand for skilled care, and increased healthcare-seeking behaviour can lead to significant reductions in neonatal mortality (24). Within this context, understanding the domiciliary newborn-care practices and healthcare-seeking for illness are of paramount importance for developing strategies, including behaviour-change communications, to prevent these deaths. More importantly, formative research on community newborn-care practices and healthcare-seeking behaviour is required to provide the foundation on which behaviour-change communication programmes can be designed and implemented (25). The research would require addressing practices of the mother, her nuclear family, traditional birth attendants, traditional healers, and formal healthcare providers and facilities.

Diarrhoea remains a highly-prevalent illness among under-five children in all parts of Bangladesh. Households seeking help from a healthcare provider overwhelmingly use the private sector in Bangladesh. The majority of children receive oral rehydration therapy (ORT); however, its use is still lower among rural populations. Gender inequities in the use of licensed care providers and purchase of antibiotics, favouring males, were identified (26). However, diarrhoea among neonates and various infectious diseases remain as major problems in Bangladesh. Compared to other developing countries, Bangladesh achieved a significant progress in the management of diarrhoea (27). Despite the notable achievement in the treatment of diarrhoeal diseases, treatment of pneumonia is yet to be improved remarkably. Zinc tablet, although targeted to the treat­ment for diarrhoea, may have its greatest benefit in reducing rates of severe pneumonia among those children who receive it consistently for diarrhoea. When given as an adjunct therapy for the treatment of diarrhoea, zinc decreases the duration and severity of the epi­sode (20, 28). In addition, supplementation of zinc for 10-14 days, given during a diarrhoeal episode, has been shown to decrease diarrhoea and ALRIs in 2-3 months following the episode (20, 28). Thus, this treatment should be emphasized in future programmes and interventions. Besides, improvements in water quality and sanitation are also needed. There are many opportunities for scal­ing up community-based measures, such as hand­washing and water purification at the point-of-use rather than waiting for large-scale engineering projects to provide water and sanitation services (27).

There is a wide-ranging neonatal mortality, along with other child mortality, by sex, residence, and division in Bangladesh that prioritizes the importance of area-specific focus. Although it is necessary to continue the existing programme to sustain the present rate and current trend, Rajshahi, Sylhet and Dhaka divisions, along with rural areas, need special consideration. Efforts to reduce child mortality also need to be strengthened so that the national target can be a reality by 2015. Emphasis should be given on the use of maternal healthcare services, including ANC, institutional delivery, delivery by medically-trained care provider, and postnatal care so that birth outcomes become better, and detection of pregnancy-related complications, neonatal childhood illness, and treatment can be provided immediately. Sylhet division and rural areas should receive top priority in this regard. Moreover, in the case of diarrhoea, Sylhet division should be covered more emphatically that has lower rates of use of services for diarrhoeal illness, especially ORS or HMS. However, in Bangladesh, as in other South Asian countries, seeking help from a care provider is primarily driven by a caretaker's expectation that his/her child requires a drug treatment as ORT has not been sufficient (29, 30). In this regard, special programmes, including targeted campaign, may be undertaken in Sylhet division and in rural areas to promote the use of ORS.

Health facility-based treatment should be encouraged for better health outcome of the ill child. Area-specific research is needed to understand health service-use behaviour so that area-specific intervention strategies can be developed to draw mothers and children into health facility. Due to cultural perceptions and other reasons, people are less likely to visit a health facility. In most cases, a traditional healer or a pharmacy plays a major role in treating childhood diseases. As a result, facility-based treatment is less-used. For the proper detection and treatment of childhood diseases, the use of professional medical care should be increased for the people who need these services.

Although there are many facilities with adequate workforce in Bangladesh to provide care to mothers and children, poor governance in the overall public health sector is a big problem. There is widespread absenteeism of doctors and paramedics at the government health centres; most government health facilities are in a state of disrepair; and the availability of drugs and medical supplies is very limited (31). More research and training is needed to understand how, where, what, and whom to train so that service providers are present at their respective assigned point. The development of a strong monitoring system is also necessary to minimize work abstinence and misuse of subsidies targeting the poor to draw them in the health centre. In addition, the current plans will not meet the demand for skilled birth attendants during the coming decade, especially if the current plan of emphasizing deliveries in the home is continued. An alternative strategy of facility-based deliveries in which the practice of skilled birth attendants as a group with close connections to upazila-level emergency obstetric care is more likely to rapidly meet the needs of mothers in Bangladesh. Another alternative strategy may be to sufficiently train the service providers, especially where the client is most likely to visit for treatment (pharmacy in rural areas). Permissions should be accorded to those who have basic institutional medical knowledge and training on health education and who can give general treatment in absence of doctors so that pharmacy-owners may work as a proxy of medically-trained doctors. If this could be done, clients might get better treatment, and the health of mother and child might be ensured to a greater extent.

Breastfeeding has a direct link to neonatal illness and nutritional status. Although breastfeeding is almost universal in Bangladesh, providing colostrums to the newborn, along with increasing duration of exclusive breastfeeding, is very crucial. Due to its thick and concentrated texture, it is believed that the baby would not digest colostrum. It is also considered to cause fever and illness of the mother if she feeds colostrum to the baby (32). In this regard, special programmes should be developed based on qualitative and quantitative studies on mothers so that interventional measures are well-understood and well-directed. Severe vitamin A deficiency has long been recognized as a potentially-lethal but preventable nutritional disease. Vitamin A is an essential nutrient needed in small amounts for normal cellular function and is especially required for the visual system, growth, and development, maintenance of epithelial cellular integrity, immune function, and reproduction (33). The current status shows that this area needs further increase in coverage of vitamin A supplementation. Promoting the production and consumption of vitamin A-rich fruits and vegetables by poor households through home-gardening should be encouraged. Although there is the Agricultural Support Services Programme, encouragement through media is also required in this regard. In Bangladesh, there have not yet been any effective measures which have sought to address the problem of vitamin A deficiency through fortification of foods. However, one of the major underlying problems, in this regard, has been the need to identify a suitable vehicle by which foods can be fortified with vitamin A and be made available to most people at risk. Recently, the United Nations Children's Fund has assessed the feasibility of vitamin A fortification and invited owners of the vegetable-oil and wheat-flour mills to explore the possibility of developing the fortification of food with vitamin A. However, it needs more focus.

Despite the primary, secondary and tertiary-level health facility, the quality of service-delivery in the overall public health sector in the country is still poor (34). Although Bangladesh has undertaken a two-pronged (community-and facility-based) approach to ensure maternal health services and has an extensive rural healthcare and family-planning service-delivery infrastructure, services have not reached most people who need these. More importantly, connection of families with the various components of the health system is lacking in Bangladesh, without which ‘access’ to healthcare remains hypothetical (27). In addition, unfavourable doctor-to-nurse ratio (which is 2:1 whereas the internationally-accepted standard is 1:3), non-availability of trained nurses and paramedics in required numbers, low coverage (especially in urban slums and inaccessible rural areas), lack of an institutional mechanism to bring the very poor and vulnerable people within the ambit of health service-delivery, negative attitudes of service providers towards the poor, and gender bias encouraging mothers to keep childbearing until they have a male child are responsible for low service-use. Besides, the use of public services is hindered by the unavailability of healthcare providers and unofficial payments (35). Thus, consideration should be given to these issues to further enrich the quality of health service and their maximum use.

Tax and donor-financed supply-side subsidies have been the main strategy for improving the access of poor people to health, nutrition, and population services in Bangladesh. The limitation of supply-side subsidies is that the target group does not receive the subsidy directly. Instead, they receive these from service providers. As a result, in the absence of an effective subsidy system, in many cases, such efforts are often poorly targeted, resulting in misuse. This is another area where monitoring should be improved. The finance may be a problem in running programmes targeting at reducing child mortality but through proper planning (i.e. budget allocation as per the need and setting up separate treatment fee for the rich and poor. Subsidy can be increased to the most vulnerable population). In addition, pursuance (i.e. finding out alternative donor agencies inside the country, pursuance of donor agencies in an effective manner globally, and prioritizing the agenda of financial assistance from different international organization regarding maternal and child healthcare) can reduce the financial burden of the health sector in Bangladesh.

The population policy of Bangladesh has a major goal to reduce infant and under-five mortality rates, along with reducing maternal and child malnutrition. To do so, different strategies were considered, including provision for maternal, child and reproductive health services through a comprehensive client-centred approach at the upazila (subdistrict) and union (lowest administrative tier) levels, safe delivery through skilled birth attendants, ensuring supply of vitamin A and other micronutrients, and prevention of malnutrition among children and pregnant women, supporting and ensuring the full coverage of child immunization (36). Although studies found a great diversity in behaviours in the use of health services, a division-wise strategy to reduce child mortality is yet to be considered at the policy level. In Sylhet, people are less likely to receive services from medically-trained service providers, and intervention is also difficult mainly because of the presence of high religiosity among its people. Besides, the environmental strategy included in the population policy did not mention any discouragement of the use of solid fuels in cooking practices so that the prevalence of ARI and pneumonia can be prevented.

## CONCLUSIONS

This paper reviewed the present status of child mortality and provided recommendations for further reduction of child mortality in Bangladesh. Overall, this study found that achievement in child mortality in Bangladesh is optimistic, especially in infant and under-five mortality but immunization is yet to achieve universal coverage as it is needed to achieve MDG 4. To improve the health of children further, some issues, such as lower use of maternal healthcare services, hazardous environmental effects on childhood illness, high malnutrition among children, shorter duration of exclusive breastfeeding practices, various child injuries leading to death and low healthcare-use for children, and strategies lacking area-wise focus on child mortality need to be considered. Neonates should be given the highest priority so that neonatal deaths contribute more to the reduction of under-five mortality in this country. Area-specific focus should be given on an emergency basis in achieving the MDG relating to under-five mortally on time. Management of financial support from inside and outside the country should also be ensured so that services provided to the most needy population, especially the rural poor, become smooth. Without proper consideration of these issues, the achievement of MDG relating to child mortality may not be possible within 2015.

## ACKNOWLEDGEMETNS

The authors are grateful to Professor David A. Sack, Department of International Health, Johns Hopkins Bloomberg School of Public Health, USA, for his valuable suggestions in finalizing the draft manuscript.
